# Highly Aggregated Structures in Aqueous Solutions from Statistical Copolymers of *N*-Vinylpyrrolidone and 2-Chloroethyl Vinyl Ether for Efficient Hydrophobic Entrapment

**DOI:** 10.3390/polym18212745

**Published:** 2026-11-04

**Authors:** Nikolaos V. Plachouras, Aineias Abedin, Stylianos Soranidis, Marinos Pitsikalis

**Affiliations:** Industrial Chemistry Laboratory, Department of Chemistry, National and Kapodistrian University of Athens, Panepistimiopolis Zografou, 15771 Athens, Greece; plachouras.v.nikolaos@gmail.com (N.V.P.); abedin.enea@gmail.com (A.A.); ssoranidis@gmail.com (S.S.)

**Keywords:** amphiphilic copolymers, statistical copolymers, poly(*N*-vinylpyrrolidone), poly(2-chloroethyl vinyl ether), self-assembly, highly aggregated structures, polymeric nanostructures, hydrophobic entrapment, curcumin, light scattering

## Abstract

The aqueous self-assembly behavior of amphiphilic statistical copolymers, composed of hydrophilic *N*-vinylpyrrolidone (NVP) and hydrophobic 2-chloroethyl vinyl ether (CEVE), was investigated. Dynamic and static light scattering (DLS and SLS) analyses revealed significant structural variations heavily dependent on the copolymer composition. Specifically, formulations with a high hydrophobic CEVE content exhibited a clear tendency to form highly aggregated systems in aqueous media. These morphological transitions and the resulting supramolecular architectures were visually confirmed via Transmission Electron Microscopy (TEM). The encapsulation capacity of these assemblies was subsequently evaluated using curcumin as a model hydrophobic guest. Despite their inherent statistical sequence, which typically lacks the distinct microphase separation observed in block copolymers, these systems successfully self-assembled and demonstrated high encapsulation efficiency. These findings indicate that such statistical copolymers constitute an effective and structurally simple platform for the entrapment of hydrophobic compounds, underscoring their potential in advanced encapsulation applications.

## 1. Introduction

The self-assembly of amphiphilic copolymers is a core concept in macromolecular science, studied systematically since 1976 [1]. This interest stems from their ability to form well-defined, stable structures in selective solvents, outperforming conventional low-molecular-weight surfactants [2]. At the molecular level, self-assembly depends on solvent quality and segment incompatibility, quantified by the Flory–Huggins interaction parameter (χ) [3]. When χ exceeds a critical threshold, the system minimizes interfacial free energy by driving the insoluble blocks to aggregate into a core [4]. While micellization occurs in various selective solvents, current research emphasizes aqueous systems to develop nanocarriers for hydrophobic drug solubilization and encapsulation [5,6]. In water, micellization is driven by the hydrophobic effect, which relies on an entropic balance [7,8,9,10,11]. The aggregation of hydrophobic segments causes a loss in conformational entropy for the polymer chains. However, this penalty is offset by a larger gain in the translational entropy of the system. This gain arises from the disruption of the structured hydration layer around the hydrophobic moieties [12]. The release of these ordered water molecules into the bulk solution increases the overall entropy, providing the primary thermodynamic driving force for micellization [8,12].

Historically, research on aqueous self-assembly has concentrated on well-defined architectures [2,13,14], primarily block copolymers [15,16,17]. The spatial segregation of their hydrophilic and hydrophobic blocks allows for predictable hydrophobic interactions and micellar morphologies [4,18,19]. To further tailor the functional properties of these nanostructures, macromolecular design has expanded to complex non-linear topologies [20,21,22]. These include star-shaped copolymers, with miktoarm architectures imposing specific topological constraints [21,23], as well as densely grafted brushes [24], giant bottlebrush block copolymers [25], dendronized structures [26,27], and multicompartment micelles [28]. Although these advanced architectures provide excellent control over self-assembly, their synthesis involves multi-step procedures, protection-deprotection chemistries, demanding purifications, and challenges in industrial scale-up [21,29,30,31,32].

In contrast to these complex topologies, linear statistical copolymers can be synthesized straightforwardly via single-step (one-pot) polymerizations [33,34]. However, their aqueous self-assembly presents a distinct thermodynamic challenge [35]. Due to the random monomer distribution along the backbone, the hydrophilic and hydrophobic segments alternate without forming discrete blocks. This lack of clear microphase separation typically hinders the formation of well-defined intermolecular micelles. Instead, the chains often favor intramolecular folding, leading to single-chain nanoparticles [36,37,38]. Because of this competing equilibrium, linear statistical copolymers have been less prominent in self-assembly studies. Nevertheless, recent reports indicate that appropriately designed random copolymers can self-assemble into uniform nanoaggregates [35,39,40,41,42,43,44]. Addressing this, the present work investigates linear amphiphilic copolymers of *N*-vinylpyrrolidone (NVP) and 2-chloroethyl vinyl ether (CEVE) that overcome the typical structural limitations of statistical sequences. As demonstrated, these systems form well-defined aggregates in water and effectively encapsulate hydrophobic guests.

The synthesis and chemical characterization of these statistical copolymers via Reversible Addition–Fragmentation chain Transfer (RAFT) polymerization were detailed in a previous report [45]. Although synthesized in a one-pot process, the large difference in monomer reactivity ratios (*r_NVP_* = 10.90 ≫ *r_CEVE_* = 0.06) dictates the formation of a gradient (pseudo-diblock) microstructure [45]. This gradient sequence is the critical feature enabling their intermolecular self-assembly. The monomer selection serves specific functional purposes. NVP acts as the hydrophilic, hemocompatible “stealth” segment, offering a leading alternative to poly(ethylene glycol) (PEG) to bypass PEG-related immunogenicity [46,47,48,49,50]. Furthermore, the lactam ring of NVP provides a strong complexation capacity [51], making PNVP suitable not only for biomedical uses but also for advanced wastewater treatment applications involving the entrapment of hydrophobic pollutants, phenolic compounds, and heavy metals [52,53,54]. CEVE provides the requisite hydrophobicity to drive aqueous self-assembly. While poly(vinyl ether)s are often associated with industrial coatings [55], PCEVE is non-toxic and suitable for biomaterials [56]. Additionally, the pendant chloroethyl groups of CEVE offer reactive sites for post-polymerization modifications [56,57]. Thus, these P(NVP-stat-CEVE) copolymers function both as autonomous self-assembling systems and as scaffolds for future complex architectures [58].

Building on our previous studies regarding the drug-loading capabilities of NVP-based systems [59,60], this work evaluates the aqueous self-assembly of the P(NVP-stat-CEVE) copolymers. The structural and physicochemical properties of the resulting nanoaggregates were characterized using Dynamic and Static Light Scattering (DLS, SLS) and Transmission Electron Microscopy (TEM). Finally, curcumin was used as a hydrophobic model drug to assess the encapsulation capacity of these systems, with loading efficiencies quantified by UV-Vis spectroscopy.

## 2. Materials and Methods

### 2.1. Synthesis and Molecular Characteristics of the P(NVP-Stat-CEVE) Statistical Copolymers

**Scheme 1:**
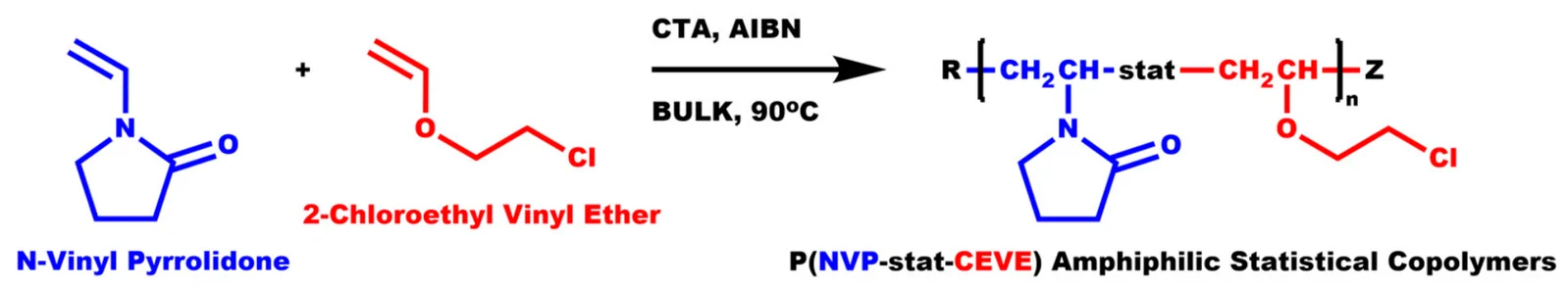
Synthesis of the P(NVP-stat-CEVE) amphiphilic statistical copolymers.

As highlighted in the Introduction, the synthesis of the amphiphilic P(NVP-stat-CEVE) statistical copolymers was accomplished via radical Reversible Addition–Fragmentation chain Transfer (RAFT) polymerization. Overcoming the traditional limitation of vinyl ethers, which typically undergo cationic polymerization, this controlled approach was successfully conducted without the requirement of any stabilizing additives. The reactions were performed in bulk at 90 °C, utilizing azobisisobutyronitrile (AIBN, Sigma-Aldrich, St. Louis, MO, USA) as the radical initiator and [(O-ethylxanthyl)methyl]benzene (synthesized in-house as described in [45]) as the chain transfer agent (CTA). Comprehensive details concerning this synthetic procedure, the experimental protocols, and the purification steps, as well as the primary molecular and therma characterization of these materials, have been reported in our previous publication [45]. The general synthetic pathway, alongside the chemical structures of the two monomers and the resulting amphiphilic statistical copolymer, is depicted in Scheme 1.

The specific macromolecular characteristics and molar compositions of the five samples utilized in the current aqueous self-assembly and drug encapsulation studies are summarized in Table 1. The corresponding Size Exclusion Chromatography (SEC) chromatograms and representative ^1^H-NMR spectra are provided in the Supplementary Materials, Figures S1 and S2. The Size Exclusion Chromatography (SEC) measurements were performed on a Waters system (Waters Corporation, Milford, MA, USA) equipped with a model 510 pump, a U6K sample injector, a Waters 401 differential refractometer, and a set of four 5 µm Styragel columns (porosity range: 500 to 600,000 Å). Calibration was performed using eight narrow polystyrene standards ranging from 2500 to 600,000 Da (Sigma-Aldrich, St. Louis, MO, USA). Chromatographic data were acquired and processed using Empower 3 software (Waters Corporation, Milford, MA, USA). For clarity throughout this work, the synthesized copolymers are designated according to their initial monomer feed molar ratios; for instance, P(NVP-stat-CEVE) 80/20 refers to a copolymer prepared with a feed composition of 80 mol% NVP and 20 mol% CEVE. As established by the kinetic and statistical analysis in our prior work [45], the disparity in monomer reactivity ratios inherently dictates a gradient or pseudo-diblock macromolecular architecture along the copolymer chain. This specific sequence distribution acts as the fundamental driving force for their spontaneous self-assembly in aqueous media, enabling the formation of distinct nanostructures.

### 2.2. Aqueous Self-Assembly and Light Scattering Characterization

#### 2.2.1. Preparation of the Micellar Dispersions

To trigger the spontaneous self-assembly of the amphiphilic macromolecules, a solvent-exchange (nanoprecipitation) protocol was applied [23,24]. To ensure precision in determining the final polymer concentration and to prevent material loss associated with mechanical agitation (e.g., adsorption onto magnetic stir bars), a rigorous gravimetric methodology was implemented throughout the entire procedure.

Initially, the copolymer samples (approximately 20 mg) were introduced into pre-weighed vials and dissolved in 5 mL of anhydrous tetrahydrofuran (THF, Sigma-Aldrich, St. Louis, MO, USA, dried and distilled over sodium metal). Although THF is a good solvent for CEVE, it generally cannot dissolve high-molecular-weight PNVP. However, as demonstrated in previous works [59,60], THF readily dissolves PVNP-based copolymers at lower molecular weights. Given the low molecular weights of the samples in this study, THF was selected to ensure complete dissolution. The solutions were then allowed to completely solubilize overnight under static conditions. Subsequently, 20 mL of ultrapure water was introduced dropwise into the THF solutions, serving as a highly selective solvent for the hydrophilic PNVP segments. The organic solvent was then quantitatively removed by mild heating at 65 °C. By sequentially recording the mass at each preparation step, the exact mass of the residual aqueous micellar dispersion was determined, allowing for the precise calculation of the primary stock solutions’ concentration (typically ~1.5 mg/mL). These stable dispersions were then appropriately diluted by weight to obtain a concentration series ranging from 0.1 up to 1.3 mg/mL for the subsequent scattering analyses.

To ensure reproducibility during the Dynamic Light Scattering (DLS) data acquisition, a measurement protocol was strictly followed for the prepared concentration series. Prior to measurement, each sample was allowed a 2 min thermal and hydrodynamic equilibration period within the spectrometer cell. Subsequently, 10 independent, consecutive 60 s measurements were recorded for every single dilution point. The final reported physical quantities represent the arithmetic mean of these 10 independent runs, with the corresponding standard deviations explicitly depicted as error bars in all plotted data, further validating the stability and homogeneity of the micellar assemblies.

#### 2.2.2. Instrumentation and Theoretical Background

Dynamic and Static Light Scattering (DLS and SLS) experiments were executed on a Brookhaven NanoBrook Omni instrument (Brookhaven Instruments Corporation, Holtsville, NY, USA), equipped with a 22 mW JDS Uniphase He-Ne laser (JDS Uniphase Corporation, Milpitas, CA, USA) emitting at a wavelength (*λ*) of 640 nm. Prior to data collection, all aqueous solutions were filtered through 0.45 µm hydrophilic PVDF syringe filters directly into the scattering cells. All measurements were conducted at 25 °C at a fixed scattering angle (*θ*) of 90°.

For the DLS analysis, the intensity–time autocorrelation functions, *g*^(2)^(*q*,*t*), were recorded using Particle Solutions software (v. 3.6.0.6376, Brookhaven Instruments Corporation, Holtsville, NY, USA) to extract the dynamic properties of the aggregates. To obtain the apparent translational diffusion coefficients (*D_app_*) and the polydispersity index (PDI) of the assemblies, the correlation data were processed using the standard cumulant expansion method [61]. The corresponding apparent hydrodynamic radii ($R_{h}^{0}$) were subsequently deduced at infinite dilution via the Stokes–Einstein relationship [62]. To effectively resolve complex or multimodal size distributions, the CONTIN Laplace inversion algorithm was additionally employed [63]. The concentration dependence of the diffusion coefficient was evaluated through the linear relation *D* = *D*_0,*app*_(1 + *k_D_c*), where *k_D_* represents the diffusion interaction parameter.

Regarding the SLS data, the structural and thermodynamic parameters of the micelles were interpreted using ZetaPlus Molecular Weight Software (v. 5.32, Brookhaven Instruments Corporation, Holtsville, NY, USA), utilizing the classic Debye formalism, namely $\frac{K_{c}}{\Delta R_{\theta }}=\frac{1}{M_{w}}+2A_{2}c$, where Δ*R_θ_* stands for the excess Rayleigh ratio relative to the solvent, *K* represents the optical constant, and *A*_2_ is the second virial coefficient, which elucidates the thermodynamic interactions between the nanostructures and the aqueous continuous phase [64,65]. The specific refractive index increments (*dn*/*dc*) essential for the evaluation of *K* were experimentally determined using a Milton Roy Chromatix KMX-16 differential refractometer (Milton Roy, Riviera Beach, FL, USA).

### 2.3. Transmission Electron Microscopy (TEM)

Morphological observation of the self-assembled nanostructures was performed using a Hitachi HT7800 transmission electron microscope (Hitachi High-Tech Corporation, Tokyo, Japan). To prepare the specimens for imaging, a 5 µL droplet of the respective aqueous micellar dispersion was deposited onto 200-mesh carbon-coated copper grids (ProSciTech Pty. Ltd., Thurnigowa, Australia). The droplets were left in contact with the grid surface for 2 min to allow adequate adsorption of the polymeric aggregates. Subsequently, the excess dispersion was carefully wicked away using Whatman 541 filter paper. The grids were then allowed to air-dry completely at ambient temperature for roughly 20 min prior to observation. Imaging was conducted in their native state, without the application of any negative staining agents, as the presence of chlorine atoms in the CEVE segments provides sufficient inherent electron contrast.

### 2.4. Encapsulation Study

#### 2.4.1. Curcumin as Model Hydrophobic Payload

To assess the encapsulation capacity and internal core dimensions of the formulated nanostructures, curcumin was utilized as a hydrophobic payload. Curcumin (Sigma-Aldrich, St. Louis, MO, USA) (diferuloylmethane) is a naturally occurring, voluminous polyphenolic compound extensively documented for its pleiotropic biological activities, including anti-inflammatory, antioxidant, and anticancer properties [66,67,68]. In the context of this study, curcumin is not exclusively employed to validate the system’s potential as a nanocarrier for biomedicine. Rather, due to its pronounced hydrophobicity, rigid conjugated backbone, and considerable molecular volume, it serves as an excellent stress-test molecule. Its successful entrapment stands as a clear proof-of-concept, directly demonstrating the high capacity of the P(NVP-stat-CEVE) scaffolds to sequester and isolate large and complex aromatic structures, phenolics, and bulky hydrophobic pollutants. Consequently, this underlines the broad applicability of these polymeric scaffolds across diverse fields, ranging from environmental technologies and water remediation to advanced macromolecular engineering and targeted delivery systems.

**Scheme 2:**
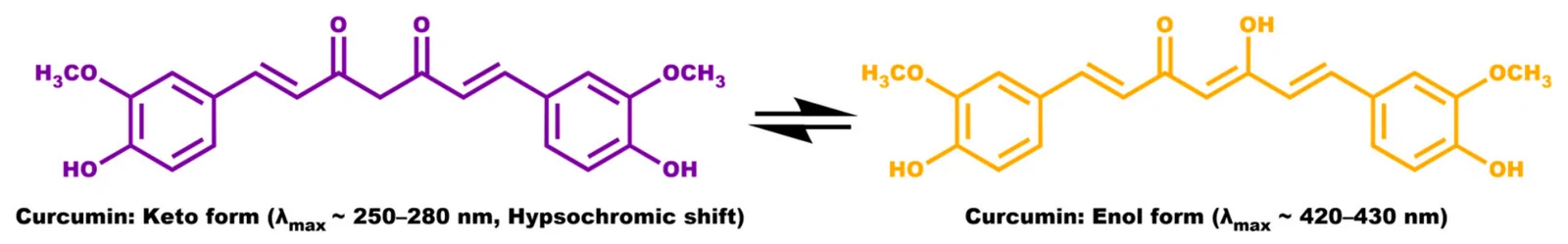
Keto–enol tautomeric equilibrium of curcumin.

From a physicochemical perspective, curcumin exists in a sensitive keto–enol tautomeric equilibrium (Scheme 2) that heavily dictates its UV-Vis absorption profile [69,70,71,72]. While the enol form characteristically absorbs in the visible spectrum near 420–430 nm, changes in solvent polarity and pH can cause spectral alterations. Additionally, strong host–guest complexation within the restricted hydrophobic environment of a micellar core can induce significant hypsochromic (blue) shifts, moving absorption maxima into the UV region (250–280 nm) due to the stabilization of the diketo form or other structurally constrained transient states [69,70,71,72]. Although the diketo conformer is frequently considered less pharmaceutically active, confirming its quantitative entrapment remains the primary objective herein, indisputably verifying the exceptional host capacity of the polymeric assemblies.

#### 2.4.2. Preparation of the Loaded Micellar Dispersions

The entrapment of curcumin within the hydrophobic domains of the P(NVP-stat-CEVE) assemblies was accomplished via a modified nanoprecipitation protocol. All solutions were handled and stored in amber glass vials to prevent photodegradation throughout the procedure. Briefly, appropriate quantities of the copolymers (approximately 20 mg) were completely dissolved in THF, followed by the addition of a concentrated curcumin solution in THF. This addition was precisely calculated to achieve three predetermined drug-to-polymer weight ratios, specifically 10%, 15%, and 20% *w*/*w* curcumin-to-polymer. Following an overnight equilibration period under static conditions to ensure the intimate mixing of the host–guest components, 20 mL of ultrapure water was introduced dropwise to induce micellization. The organic solvent was then evaporated via gentle heating at 65 °C, and the exact final concentrations were determined gravimetrically. To obtain an accurate baseline for subsequent spectrophotometric analyses, an identical protocol was performed simultaneously without the addition of curcumin, yielding corresponding empty (blank) micellar solutions.

#### 2.4.3. Determination of Drug Loading Capacity and Efficiency

The quantitative evaluation of the entrapped curcumin was executed utilizing a Perkin Elmer Lambda 650 UV-Vis spectrophotometer (PerkinElmer, Waltham, MA, USA), operating across a wavelength range of 250 to 800 nm at 25 °C. Standard calibration curves were initially constructed from curcumin solutions of known concentrations in chloroform (CHCl_3_, Sigma-Aldrich, St. Louis, MO, USA) [69]. By measuring the absorbance of the loaded polymeric solutions against their respective blank (empty micelle) dispersions, the exact mass of the encapsulated payload was determined. Subsequently, the % Drug Loading Capacity (%DLC) and % Drug Loading Efficiency (%DLE) were calculated according to the following established theoretical equations derived from established micellar characterization methodologies [5,73,74]:

(1) $$\%\;\mathrm{Drug}\;\mathrm{Loading}\;\mathrm{Capacity}\left(\%\mathrm{DLC}\right)=\frac{\mathrm{mass}\;\mathrm{of}\;\mathrm{loaded}\;\mathrm{drug}}{\mathrm{mass}\;\mathrm{of}\;\mathrm{polymer}}\times 100$$

(2) $$\%\;\mathrm{Drug}\;\mathrm{Loading}\;\mathrm{Efficiency}\left(\%\mathrm{DLE}\right)=\frac{\mathrm{mass}\;\mathrm{of}\;\mathrm{loaded}\;\mathrm{drug}}{\mathrm{mass}\;\mathrm{of}\;\mathrm{drug}\;\mathrm{in}\;\mathrm{feed}}\times 100$$

## 3. Results and Discussion

### 3.1. Aqueous Self-Assembly and Nanostructural Characterization

**Figure 1:**
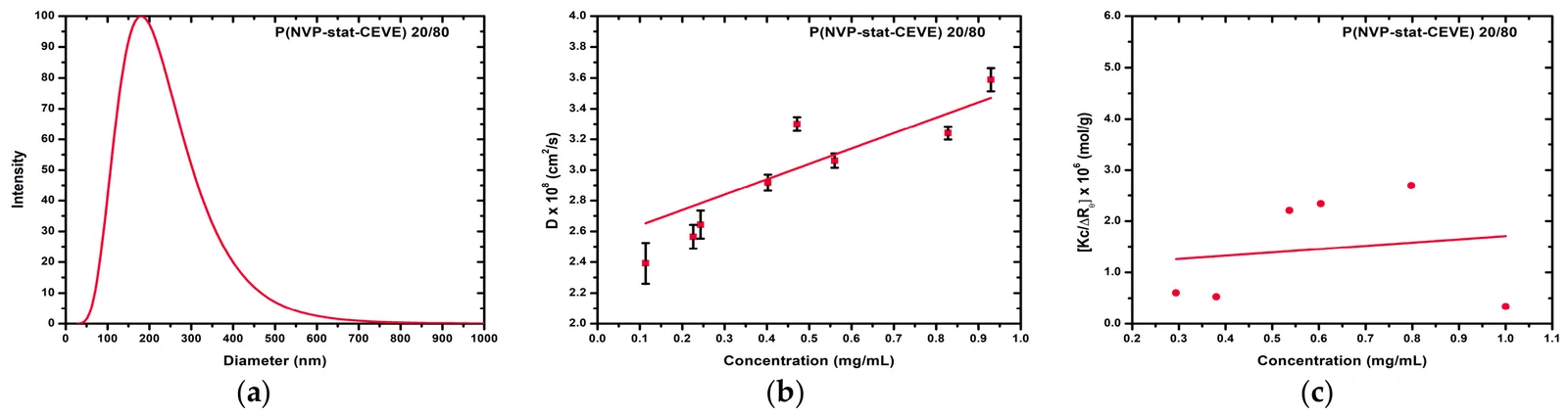
Physicochemical characterization of sample 20/80 in aqueous media: (**a**) CONTIN plot at *C* = 0.226 mg/mL with a PDI of 0.22; (**b**) apparent diffusion coefficient vs. concentration plot; and (**c**) SLS plot.

**Figure 2:**
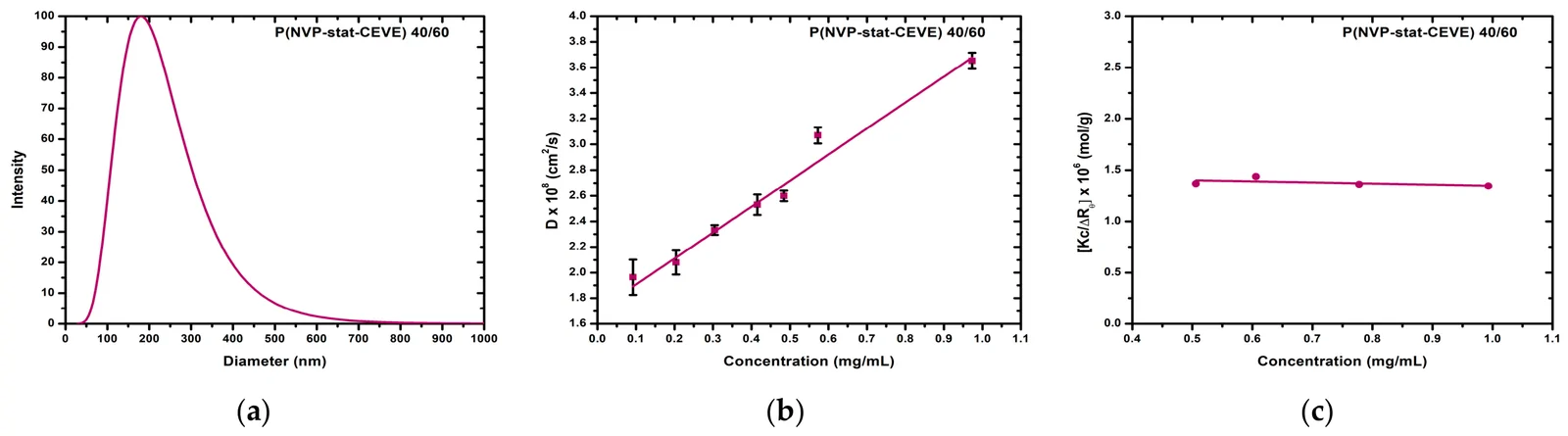
Physicochemical characterization of sample 40/60 in aqueous media: (**a**) CONTIN plot at *C* = 0.204 mg/mL with a PDI of 0.29; (**b**) apparent diffusion coefficient vs. concentration plot; and (**c**) SLS plot.

**Figure 3:**
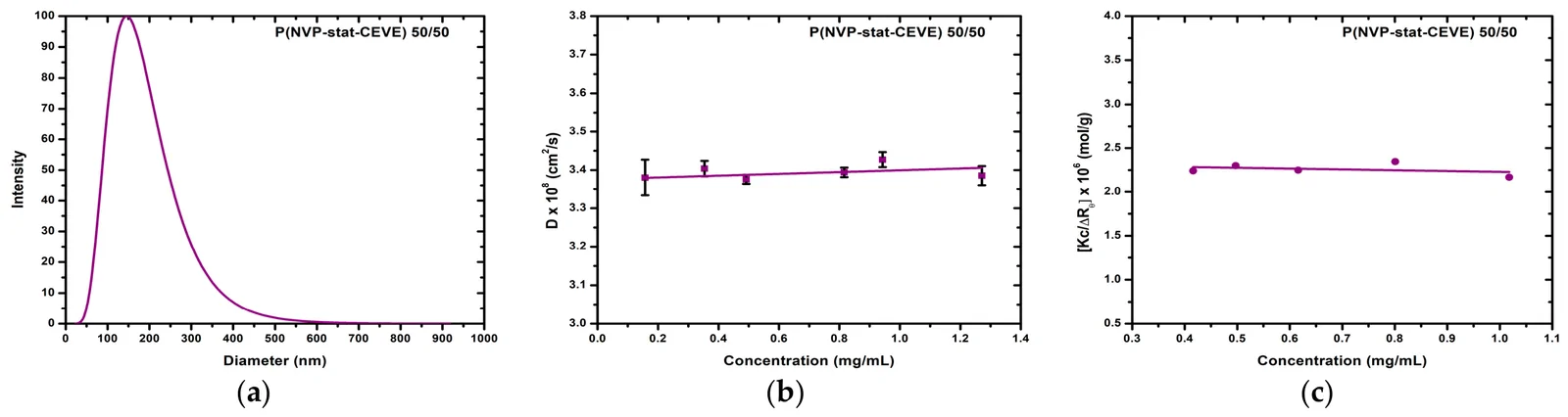
Physicochemical characterization of sample 50/50 in aqueous media: (**a**) CONTIN plot at *C* = 0.492 mg/mL with a PDIA of 0.21; (**b**) apparent diffusion coefficient vs. concentration plot; and (**c**) SLS plot.

**Figure 4:**
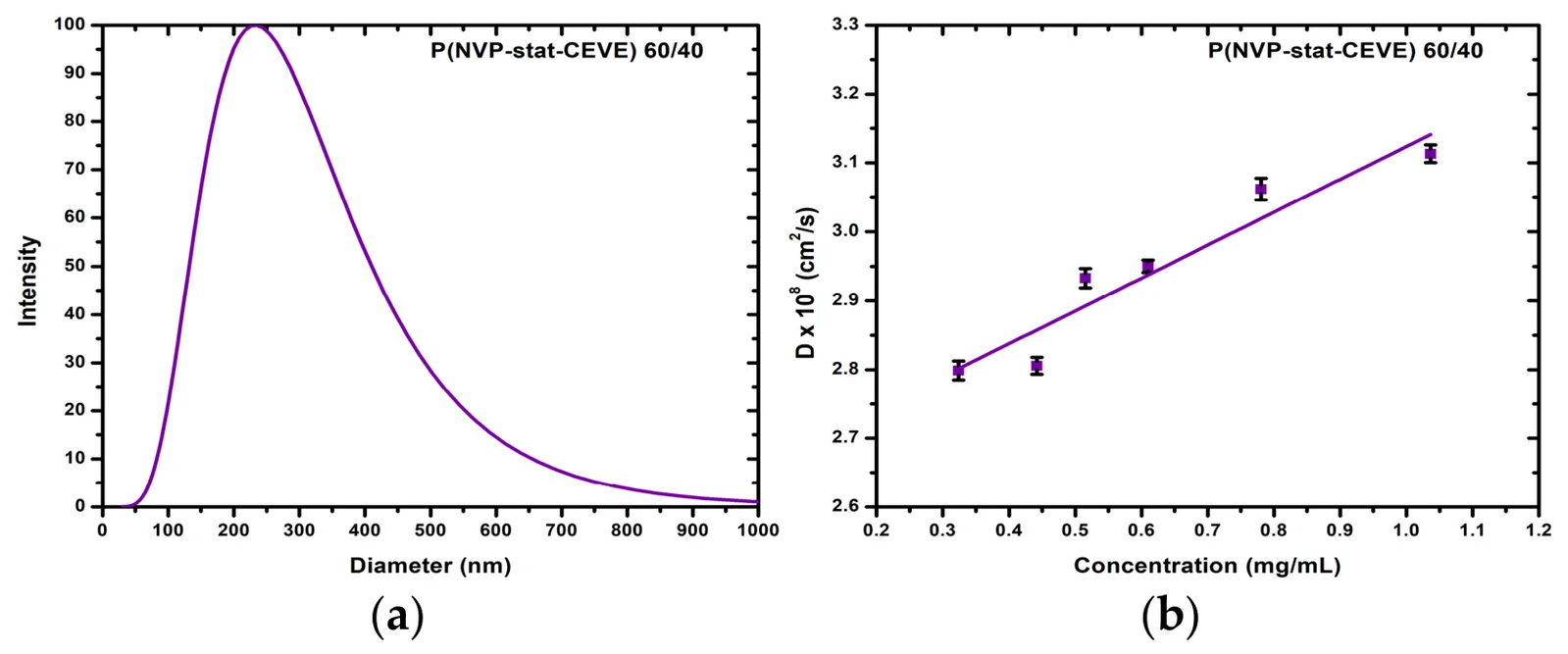
Physicochemical characterization of sample 60/40 in aqueous media: (**a**) CONTIN plot at *C* = 0.324 mg/mL with a PDI of 0.21; (**b**) apparent diffusion coefficient vs. concentration plot.

**Figure 5:**
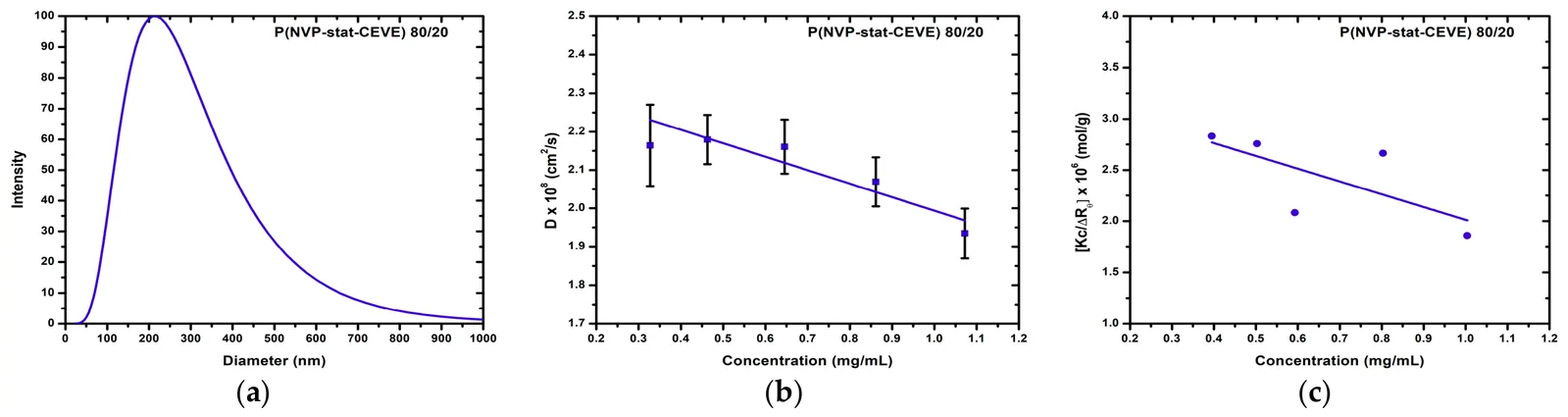
Physicochemical characterization of sample 80/20 in aqueous media: (**a**) CONTIN plot at *C* = 0.462 mg/mL with a PDI of 0.31; (**b**) apparent diffusion coefficient vs. concentration plot; and (**c**) SLS plot.

The aqueous self-assembly of the amphiphilic statistical copolymers was evaluated by DLS and SLS. Driven by the large difference in monomer reactivity ratios (*r_NVP_* = 10.90 ≫ *r_CEVE_* = 0.06), the macromolecular chains adopt a gradient sequence distribution that dictates their micellization behavior. The resulting hydrodynamic and thermodynamic parameters (Table 2 and Table 3) show a strong dependence of the assembly structure on both the hydrophobic CEVE content and the solution concentration. The nanostructural characterization is depicted in Figure 1, Figure 2, Figure 3, Figure 4 and Figure 5. For each sample, the respective figure comprises three panels presenting the CONTIN size distribution analysis (a), the apparent diffusion coefficient versus concentration dependence (b), and the single-angle (90°) SLS plot (c). To support the DLS measurements, the corresponding autocorrelation functions for the representative CONTIN plots are provided in the Supplementary Materials (Figure S3).

The high mole fraction of CEVE (34.0 mol%) for the 20/80 copolymer, which is the most hydrophobic copolymer, drives the formation of compact primary micelles. SLS analysis yielded the highest aggregation number of the copolymer series for this sample (*N_w_* = 173), reflecting the existence of a dense micellar core packing that reduces the hydrodynamic radius at infinite dilution (*R_h_*_,0_) to 96.60 nm. Both the diffusion interaction parameter (*k_d_* = 395.36 cm^3^/g) and second virial coefficients (*A*_2_) are positive, pointing to effective steric stabilization by the PNVP corona. Due to these inter-micellar repulsions, the hydrodynamic volume decreases as the solution concentration increases (Figure 1a–c).

As the CEVE fraction decreases in the 40/60 sample (11.4 mol% CEVE), the micellar core structure becomes looser, leading to a drop in the aggregation number (*N_w_* = 67.4) alongside an increase in the hydrodynamic radius. Due to the pseudo-diblock nature of the chains, the aggregates exhibit extensive swelling, resulting in the maximum *R_h_*_,0_ value of the series (143.82 nm). This system displays the highest positive *k_d_* value (1188.51 cm^3^/g), pointing to strong inter-micellar repulsions that cause the swollen assemblies to compress at higher concentrations (Figure 2a–c).

For the 50/50 sample (9.8 mol% CEVE), where *R_h_*_,0_ = 72.63 nm, and *N_w_* = 26.9, the *k_d_* coefficient decreases to a near-zero value of 6.94 cm^3^/g. The *k_d_* value is related to the second virial coefficient (*A*_2_) through the equation *k_d_* = 2*A*_2_*M* − *k_f_* − *u*, where *M* is the molecular weight, *k_f_* is the concentration dependence of the friction coefficient, and *u* is the polymer’s partial specific volume. Therefore, low *k_d_* values simply imply that thermodynamic and hydrodynamic contributions are overbalanced across the measured concentration range (Figure 3a–c).

Further reduction in the hydrophobic content in the 60/40 sample (4.9 mol% CEVE) yields loose assemblies with an *R_h_*_,0_ of 92.61 nm and weakly repulsive interactions (*k_d_* = 179.76 cm^3^/g), as determined by DLS measurements (Figure 4a,b).

Finally, the highly hydrophilic 80/20 sample (97.5 mol% NVP, 2.5 mol% CEVE) presents the lowest aggregation number (*N_w_* = 14.7) but maintains a large hydrodynamic volume (*R_h_*_,0_ = 104.51 nm) due to the hydration of the PNVP segments. Even with the extremely low fraction of hydrophobic CEVE units (2.5 mol%) randomly dispersed among the abundant hydrophilic NVP units, the system retains its capacity for self-assembly. Notably, the *k_d_* value becomes negative (−150.18 cm^3^/g). This indicates that attractive intermolecular interactions, primarily through hydrogen bonding, predominate. Consequently, at higher concentrations, these pseudo-diblock networks form transient bridges, leading to a gradual increase in their apparent size (Figure 5a–c). The absence of a rigid, compact core in these high-NVP assemblies, combined with their extensive swelling, implies increased free volume. This loose architecture provides a suitable microenvironment for accommodating hydrophobic guest molecules, which is evaluated in the subsequent encapsulation studies.

### 3.2. Morphological Characterization via Transmission Electron Microscopy (TEM)

**Figure 6:**
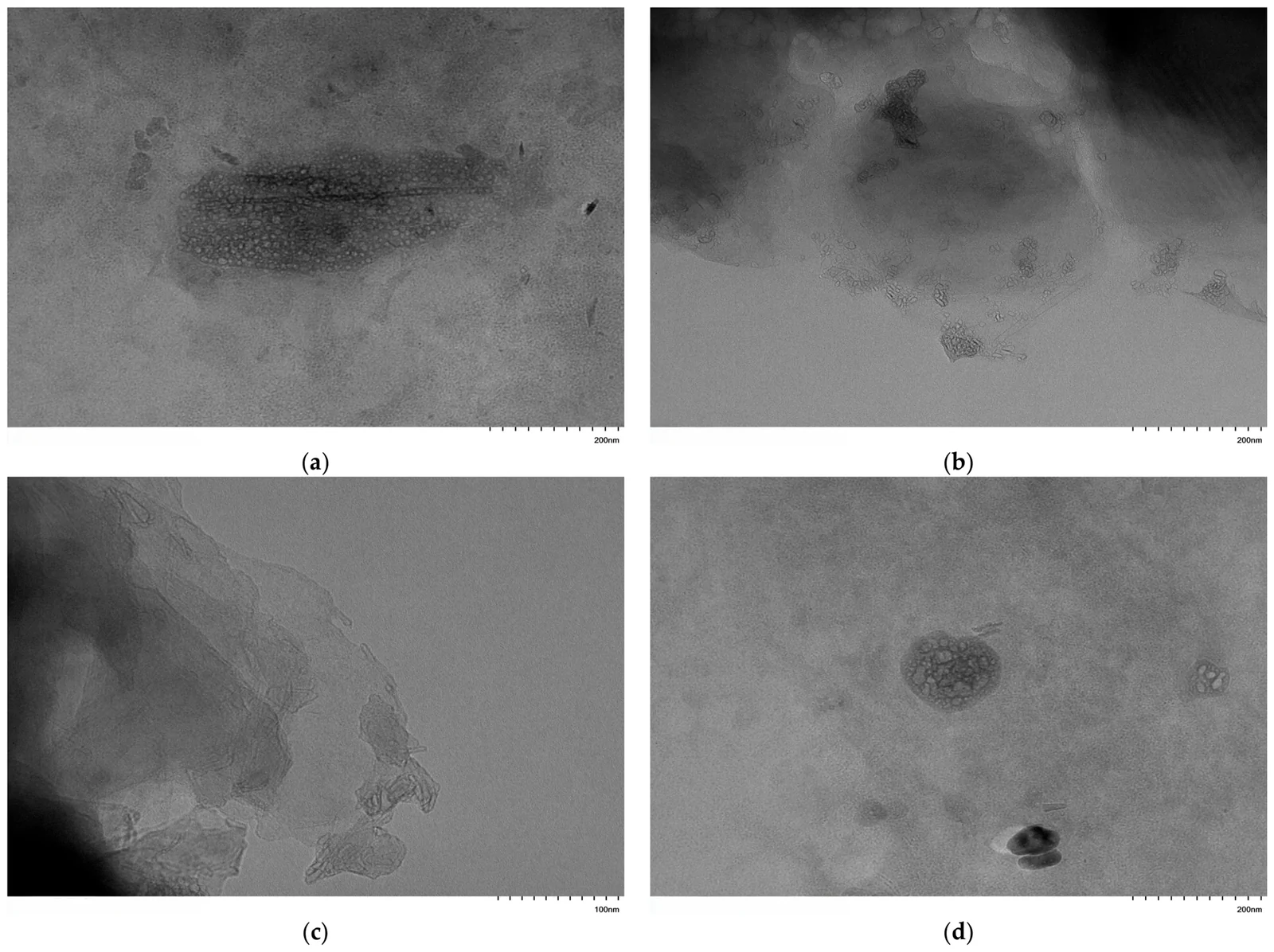
Morphological characterization of the P(NVP-stat-CEVE) assemblies via Transmission Electron Microscopy (TEM): (**a**) 80/20; (**b**) 50/50; (**c**) 40/60; (**d**) 20/80.

To visually assess the supramolecular structures inferred from the light scattering analyses, the morphology of the P(NVP-stat-CEVE) assemblies was investigated using Transmission Electron Microscopy (TEM). The selected micrographs of the representative samples, evaluated in their dry state, are presented in Figure 6a–d. The morphological progression reflects the influence of the CEVE molar content on the self-assembly mechanism.

For the highly hydrophilic P(NVP-stat-CEVE) 80/20 formulation, TEM imaging reveals an extended, loosely connected, “worm-like” network lacking a distinct, segregated core (Figure 6a). This open morphology supports the light scattering data discussed in Section 3.1, which indicated a low aggregation number and strong attractive inter-chain associations. The minimal hydrophobic content acts as sparse physical crosslinking points, resulting in a swollen, water-permeable macromolecular network rather than discrete micellar entities.

As the hydrophobic fraction increases in the 50/50 sample, the system undergoes structural contraction, as predicted by the reduced hydrodynamic radius. The corresponding micrograph (Figure 6b) displays an interconnected, sponge-like porous network. This intermediate architecture demonstrates that while the system begins to contract due to increased hydrophobic interactions, it retains sufficient free volume and a non-compact core. This visual evidence aligns with the Theta-like conditions established previously by the near-zero *k_d_* value.

A distinct morphological transition is observed in the 40/60 assembly. Despite the higher hydrophobic content compared to the 50/50 sample, the TEM image (Figure 6c) reveals a large, highly porous aggregate structure without well-defined boundaries. This visual observation is consistent with the maximum hydrodynamic dimensions and the strong inter-micellar repulsions determined by DLS. The gradient sequence of the chains hinders complete hydrophobic collapse, leading to a severely swollen architecture.

Conversely, the most hydrophobic formulation, P(NVP-stat-CEVE) 20/80, exhibits a completely distinct morphology. TEM analysis (Figure 6d) confirms the formation of denser and more compact polymeric assemblies. The size of these compact domains visually correlates with the hydrodynamic diameter obtained via DLS. This hard-sphere morphology physically validates the high aggregation number and effective steric stabilization discussed earlier, indicating that the higher CEVE fraction provides sufficient driving force for the complete collapse of the chains into a tightly packed, segregated hydrophobic core.

### 3.3. Curcumin Encapsulation and UV-Vis Spectroscopic Analysis

**Figure 7:**
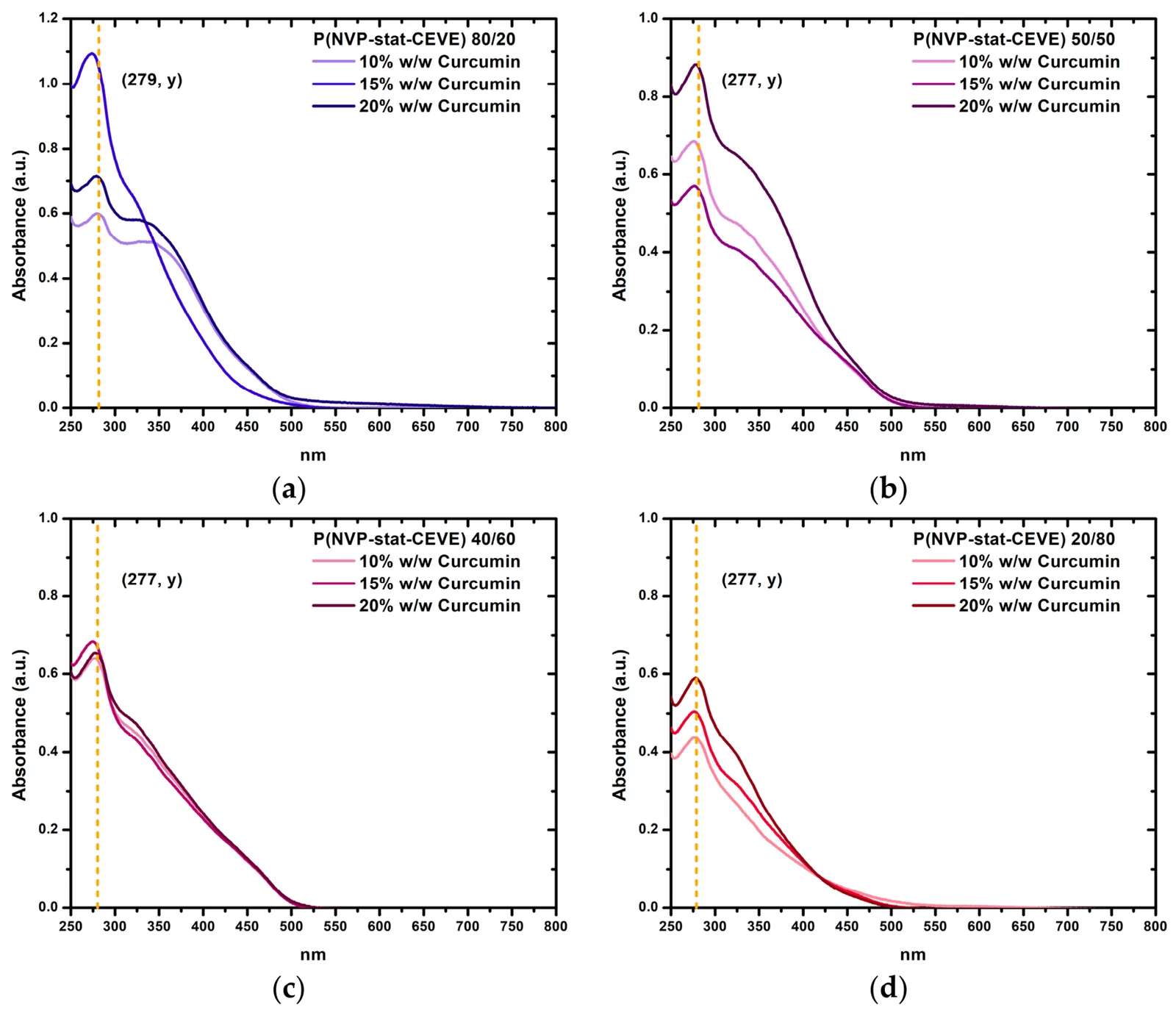
Overlay UV-vis absorption spectra of the encapsulated curcumin within the aqueous micellar solutions of the P(NVP-stat-CEVE) statistical copolymers: (**a**) 80/20, (**b**) 50/50, (**c**) 40/60, and (**d**) 20/80. The spectra illustrate the concentration-dependent absorbance across the three initial drug-to-polymer feed ratios (10%, 15%, and 20% *w*/*w*). The dashed vertical line denotes the characteristic absorption maximum of the encapsulated diketo tautomer.

The encapsulation of the highly hydrophobic model drug curcumin within the amphiphilic statistical copolymers was investigated at three initial drug-to-polymer feed ratios (10%, 15%, and 20% *w*/*w*). UV-Vis spectroscopy was employed to evaluate the optical properties and confirm the successful entrapment of the drug. The overlay spectra for each sample are presented in Figure 7, illustrating the concentration-dependent absorbance. The isolated spectra corresponding to individual loadings are provided in the Supplementary Materials (Figures S6–S9).

Curcumin exhibits keto–enol tautomerism, with its equilibrium and corresponding absorption maxima being sensitive to the local microenvironment [69]. During the construction of the calibration curve in chloroform (Figures S4 and S5 and Table S1, Supplementary Materials), the diketo tautomer showed its characteristic *π* → π^*^ transition maximum at 264 nm. Across all encapsulated samples, this peak exhibited a bathochromic (red) shift, stabilizing in the range of 274–279 nm (Table 4). This solvatochromic shift is a recognized optical response of curcumin to changes in solvent polarity and matrix interactions [69,75,76]. In this system, it serves as direct spectroscopic evidence of the drug’s incorporation into the highly hydrated polymeric network. Within this constrained microenvironment, the dielectric constant is altered, and new hydrogen-bonding interactions are established between the diketo groups of curcumin, the permeating aqueous medium, and the NVP units [77,78,79]. To avoid calculation errors associated with measuring on the spectral slope, the drug concentration was calculated by correlating the shifted local absorption maxima (~277 nm) directly to the diketo maximum of the calibration curve (264 nm), assuming a comparable molar extinction coefficient.

The quantitative encapsulation results, including the exact absorption maxima (*λ_max_*), % Drug Loading Capacity (%DLC), and Drug Loading Efficiency (%DLE), are summarized in Table 4.

The encapsulation data reveal a distinct trend regarding the hydrophilicity of the carrier and the balance between hydrophobic domains and secondary intermolecular forces [5,6,80,81]. Theoretically, a highly hydrophobic drug like curcumin would favor a core predominantly composed of hydrophobic units (CEVE). However, the most hydrophobic sample, P(NVP-stat-CEVE) 20/80, demonstrated the lowest encapsulation performance, yielding a maximum DLC of 6.46% and a DLE of 48.17%. This result is consistent with the light scattering findings and literature on micellar drug loading [73,80,82,83,84]. As established in Section 3.1, the 20/80 sample forms highly compact micelles with dense core packing. In these strongly segregated systems, cohesive polymer–polymer associations within the hydrophobic domains predominate over polymer–drug interactions [80,83,84,85,86]. This tight architecture and reduced chain mobility limit the available free spatial volume, causing steric hindrance that prevents the bulky curcumin molecules from penetrating and stabilizing within the micellar interior [82,83,84,86,87].

In contrast, the highly hydrophilic sample, P(NVP-stat-CEVE) 80/20, proved to be the most efficient carrier. At a 15% *w*/*w* initial feed ratio, it achieved a DLE of 88.37% and a DLC of 13.53%. This behavior aligns with the morphological insights previously obtained via SLS and DLS. Rather than forming rigid cores, the 80/20 sample adopts a swollen, loose architecture driven by inter-chain associations (as indicated by its negative *k_d_* value), where CEVE segments form dispersed hydrophobic microdomains within a hydrated matrix [35,88]. This expanded gradient network provides sufficient spatial volume for the large curcumin molecules to penetrate. While these accessible hydrophobic domains drive the initial drug partitioning, overall encapsulation efficiency depends on a synergy with other non-covalent forces [81,84,85,87,89]. Concurrently, the abundant NVP structural units act as hydrogen-bond acceptors via lactam carbonyl interactions with the phenolic hydroxyls of curcumin and provide dipole–dipole interaction sites, effectively anchoring the drug within the interior matrix and preventing drug-induced phase separation [77,78,89,90].

An apparent saturation effect was observed across all formulations as the initial drug feed increased. While 10% and 15% *w*/*w* feed ratios yielded optimal efficiency—particularly for the 80/20 and 40/60 assemblies—the introduction of a 20% *w*/*w* feed ratio led to a noticeable decline in DLE for all samples. This indicates that the loading capacity of the swollen micellar cores is reached, resulting in the macroscopic precipitation of the excess non-encapsulated drug. Intermediate samples such as the 40/60, despite displaying the largest initial dimensions at infinite dilution, exhibited moderate encapsulation efficiencies. As discussed in Section 3.1, this specific formulation possesses the highest thermodynamic parameter (*k_d_*) of the series. This strong repulsive behavior implies severe micellar compression upon increasing concentration, potentially squeezing out the entrapped drug during the self-assembly process. Thus, a 15% *w*/*w* feed ratio within the highly hydrated P(NVP-stat-CEVE) 80/20 assemblies was identified as the optimal formulation for maximizing the curcumin payload.

## 4. Conclusions

Water acts as a highly selective solvent for the P(NVP-stat-CEVE) amphiphilic statistical copolymers, driving their spontaneous self-assembly into defined nanostructures. Micellization was achieved despite the lack of discrete microphase separation, occurring even in the 80/20 formulation, which contains a minimal fraction of hydrophobic CEVE units. The investigation demonstrated that the resulting supramolecular architecture depends directly on the copolymer composition. As the hydrophobic CEVE percentage increases, the assemblies transition from swollen, extended pseudo-diblock networks to compact, hard-sphere micelles.

Curcumin was employed as a bulky, rigid hydrophobic model compound to evaluate the encapsulation capacity of these assemblies. The highly swollen, loose core of the hydrophilic 80/20 copolymer proved to be the most effective matrix, yielding the highest drug loading capacities. This indicates that the extended, free-volume-rich architecture inherent to these gradient sequences facilitates the accommodation of large hydrophobic molecules, bypassing the steric hindrance encountered in tightly packed micellar cores.

Beyond their effective encapsulation performance, the one-pot synthetic accessibility of these statistical copolymers provides a practical advantage over demanding block architectures. Coupled with the preservation of the reactive pendant chlorine atoms for future modifications, these macromolecules offer a highly adaptable structural scaffold. Ultimately, their demonstrated ability to solubilize and entrap bulky hydrophobic molecules establishes them as versatile nanocarriers for targeted solubilization and encapsulation applications in aqueous media.

## Figures and Tables

**Table 1:** Molecular characteristics of the amphiphilic P(NVP-stat-CEVE) statistical copolymers.

P(NVP-Stat-CEVE) (Feed Composition)	M_n_ × 10^3^ (Daltons) ^1^	M_w_ × 10^3^ (Daltons) ^1^	Đ (M_w_/M_n_) ^1^	mol% NVP ^2^	mol% CEVE ^2^
80/20	11.6	20.8	1.80	97.50	2.50
60/40	6.9	11.2	1.76	95.10	4.90
50/50	9.7	16.0	1.65	90.20	9.80
40/60	6.7	10.2	1.40	88.60	11.40
20/80	3.4	5.2	1.51	66.00	34.00

^1^ Derived by Size Exclusion Chromatography (SEC) in CHCl_3_. ^2^ Derived by ^1^H-NMR in CDCl_3_.

**Table 2:** DLS data for the P(NVP-stat-CEVE) copolymers in aqueous media.

Sample	D_0_ × 10^8^ (cm^2^/s) ^1^	k_d_ (cm^3^/g) ^2^	R_h,0_ (nm) ^3^	PDI ^4^
80/20	2.35 ± 0.12	−150.18	104.51	0.34 ± 0.01
60/40	2.65 ± 0.04	179.76	92.61	0.23 ± 0.01
50/50	3.38 ± 0.04	6.95	72.63	0.20 ± 0.01
40/60	1.70 ± 0.11	1188.51	143.82	0.29 ± 0.01
20/80	2.54 ± 0.14	395.36	96.60	0.24 ± 0.01

^1^ Apparent diffusion coefficient at infinite dilution. ^2^ Hydrodynamic interaction parameter. ^3^ Hydrodynamic radius at infinite dilution. ^4^ Polydispersity index. Note: The error reported for D_0_ represents the Standard Error (SE) of the intercept derived from the linear regression analysis, whereas the error for PDI corresponds to the Standard Error of the Mean (SEM).

**Table 3:** SLS data for the P(NVP-stat-CEVE) copolymer micellar assemblies in aqueous media.

Sample	(M_w_)_mic_ × 10^3^ (Daltons) ^1^	(A_2_)_mic_ (cm^3^mol/g^2^) ^2^	N_w_ ^3^
80/20	306	−6.25 × 10^−4^	14.7
50/50	431	−4.60 × 10^−5^	26.9
40/60	687	−5.31 × 10^−5^	67.4
20/80	900	3.00 × 10^−4^	173

^1^ Weight-average apparent micellar molecular weight. ^2^ Second virial coefficient. ^3^ Aggregation number.

**Table 4:** Results of curcumin encapsulation within the highly swollen micellar structures formed in aqueous solutions by the P(NVP-stat-CEVE) statistical copolymers.

Sample	Curc. ^1^ (% *w*/*w*)	*λ_max_* ^2^ (nm)	A ^3^ (a.u.)	Pol. ^4^ (μg/mL)	DIF ^5^ (μg/mL)	LD ^6^ (μg/mL)	DLC (%)	DLE (%)
80/20	10	279	0.60	168.51	15.57	11.98	7.11	76.98
	15	274	1.09	171.13	26.21	23.16	13.53	88.37
	20	279	0.71	172.72	33.35	14.59	8.45	43.73
50/50	10	276	0.69	163.00	17.04	13.93	8.55	81.76
	15	277	0.57	161.76	24.63	11.31	6.99	45.93
	20	277	0.88	193.69	32.87	18.40	9.50	55.99
40/60	10	277	0.64	171.75	16.66	12.91	7.52	77.49
	15	276	0.68	172.47	27.08	13.88	8.05	51.26
	20	278	0.66	173.66	32.26	13.23	7.62	41.01
20/80	10	277	0.44	179.82	17.29	8.33	4.63	48.17
	15	276	0.50	176.82	26.36	9.83	5.56	37.28
	20	278	0.59	182.16	33.07	11.76	6.46	35.57

^1^ Targeted curcumin feed ratio. ^2^ Wavelength of maximum absorbance. ^3^ Absorbance at *λ_max_*. ^4^ Concentration of the polymer stock solutions in H_2_O. ^5^ Concentration of the drug in feed. ^6^ Concentration of the loaded drug.

## Data Availability

The original contributions presented in this study are included in the article/Supplementary Materials. Further inquiries can be directed to the corresponding author.

## Supplementary Materials

The following supporting information can be downloaded at https://www.mdpi.com/article/10.3390/polym18212745/s1, Figure S1. Size Exclusion Chromatography (SEC) elution chromatograms in chloroform for the P(NVP-stat-CEVE) copolymers (80/20, 60/40, 50/50, 40/60, and 20/80). Figure S2. Representative ^1^H-NMR spectra in CDCl3 of the P(NVP-stat-CEVE) statistical copolymers: (a) 80/20; (b) 60/40; (c) 50/50; (d) 40/60; and (e) 20/80. The copolymer’s actual composition was calculated from the portions of characteristic signals assigned to the NVP and CEVE monomer units, utilizing portions of protons A and B for NVP and portions of protons C, D, and E for CEVE, as shown in the spectra. Figure S3. Intensity time autocorrelation functions derived from Dynamic Light Scattering (DLS) measurements in aqueous media for the P(NVP-stat-CEVE) statistical copolymers: (a) 80/20 C= mg/mL; (b) 60/40 at C = 0.324 mg/mL; (c) 50/50 at C = 0.492 mg/mL; (d) 40/60 at C = 0.204 mg/mL; and (e) 20/80 at C = 0.226 mg/mL. Figure S4. Individual UV-Vis absorption spectra of curcumin in chloroform (CHCl_3_) corresponding to the seven prepared concentration levels used for the calibration process: (a) C_1_ = 0.52 μg/mL; (b) C_2_ = 1.40 μg/mL; (c) C_3_ = 2.36 μg/mL; (d) C_4_ = 3.28 μg/mL; (e) C_5_ = 4.23 μg/mL; (f) C_6_ = 5.25 μg/mL; and (g) C_7_ = 6.10 μg/mL. The dashed vertical line denotes the theoretical absorption maximum of the diketo tautomer at 264 nm. Table S1. Concentration and corresponding absorbance values of curcumin in chloroform at λ_max_ = 264 nm, utilized for the construction of the calibration curve. Figure S5. Construction of the curcumin calibration curve in chloroform: (a) overlay of the UV-Vis absorption spectra for the five valid concentration levels (C_2_, C_3_, C_5_, C_6_, and C_7_); (b) the resulting linear calibration curve plotting absorbance at 264 nm versus curcumin concentration, exhibiting a high correlation coefficient (*R*^2^ = 0.9916). Figure S6. Isolated UV-Vis absorption spectra of the encapsulated curcumin within the aqueous micellar solutions of the P(NVP-stat-CEVE) 80/20 sample at three initial drug-to-polymer feed ratios: (a) 10% *w*/*w*; (b) 15% *w*/*w*; and (c) 20% *w*/*w*. The dashed vertical line denotes the characteristic absorption maximum of the encapsulated diketo tautomer. Figure S7. Isolated UV-Vis absorption spectra of the encapsulated curcumin within the aqueous micellar solutions of the P(NVP-stat-CEVE) 50/50 sample at three initial drug-to-polymer feed ratios: (a) 10% *w*/*w*; (b) 15% *w*/*w*; and (c) 20% *w*/*w*. The dashed vertical line denotes the characteristic absorption maximum of the encapsulated diketo tautomer. Figure S8. Isolated UV-Vis absorption spectra of the encapsulated curcumin within the aqueous micellar solutions of the P(NVP-stat-CEVE) 40/60 sample at three initial drug-to-polymer feed ratios: (a) 10% *w*/*w*; (b) 15% *w*/*w*; and (c) 20% *w*/*w*. The dashed vertical line denotes the characteristic absorption maximum of the encapsulated diketo tautomer. Figure S9. Isolated UV-Vis absorption spectra of the encapsulated curcumin within the aqueous micellar solutions of the P(NVP-stat-CEVE) 20/80 sample at three initial drug-to-polymer feed ratios: (a) 10% *w*/*w*; (b) 15% *w*/*w*; and (c) 20% *w*/*w*. The dashed vertical line denotes the characteristic absorption maximum of the encapsulated diketo tautomer.
