# Supplementary material for: Highly Aggregated Structures in Aqueous Solutions from Statistical Copolymers of N-Vinylpyrrolidone and 2-Chloroethyl Vinyl Ether for Efficient Hydrophobic Entrapment
Source: Polymers (Basel). 2026 Nov 4;18(21):2745. doi: 10.3390/polym18212745 (PMC13766001; doi:10.3390/polym18212745)
Supplement: Supplementary file 1 [file polymers-18-02745-s001.zip › polymers-4646014-supplementary.pdf]

Highly Aggregated Structures in Aqueous Solutions from Statistical Copolymers of *N*-Vinylpyrrolidone and 2-Chloroethyl Vinyl Ether for Efficient Hydrophobic Entrapment

Nikolaos V. Plachouras , Aineias Abedin , Stylianos Soranidis and Marinos Pitsikalis *

Industrial Chemistry Laboratory, Department of Chemistry, National and Kapodistrian University of Athens, Panepistimiopolis Zografou, 15771 Athens, Greece; plachouras.v.nikolaos@gmail.com (N.V.P.); abedin.enea@gmail.com (A.A.); ssoranidis@gmail.com (S.S.)

* Correspondence: pitsikalis@chem.uoa.gr; Tel.: +30-210-727-4768

Supporting Information Section

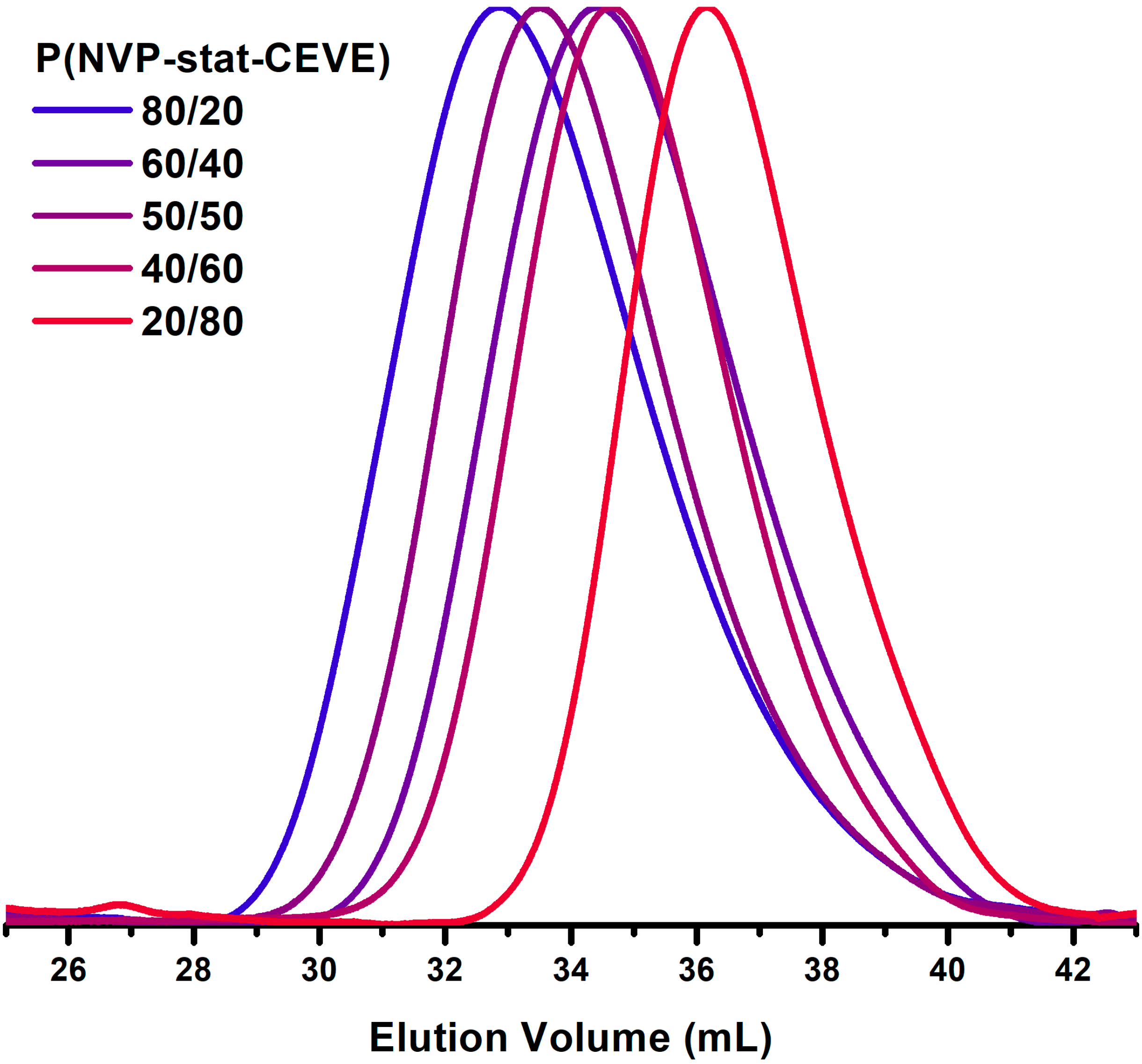

Figure S1. Size Exclusion Chromatography (SEC) elution chromatograms in chloroform for th P(NVP-stat-CEVE) copolymers (80/20, 60/40, 50/50, 40/60, and 20/80).

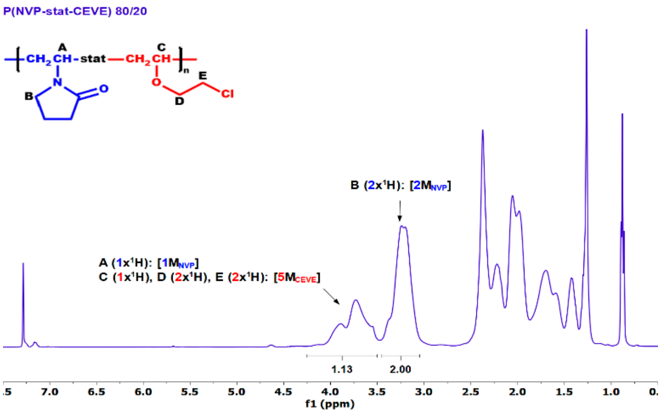

(a)

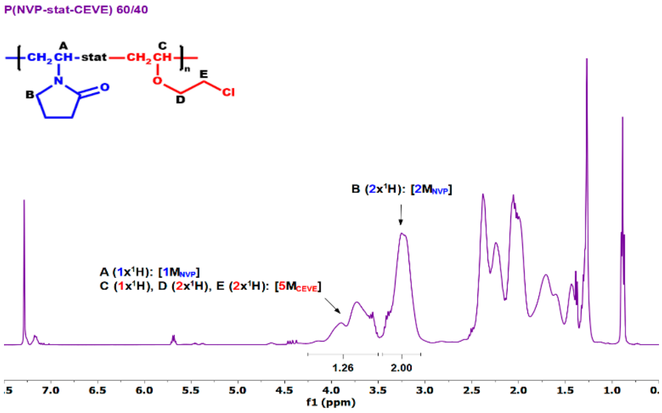

(b)

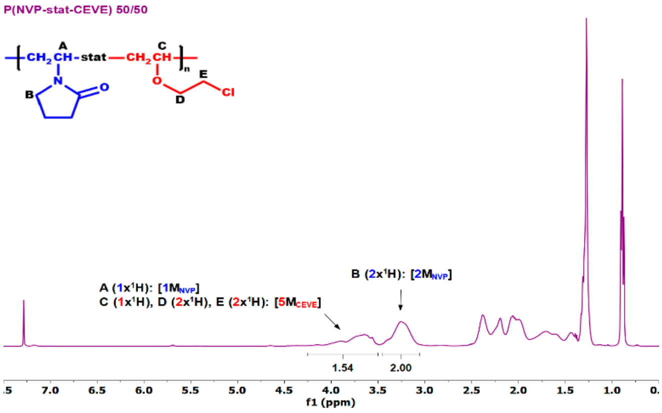

(c)

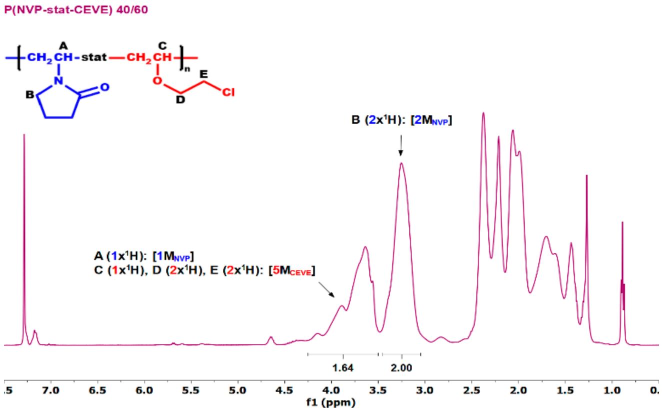

(d)

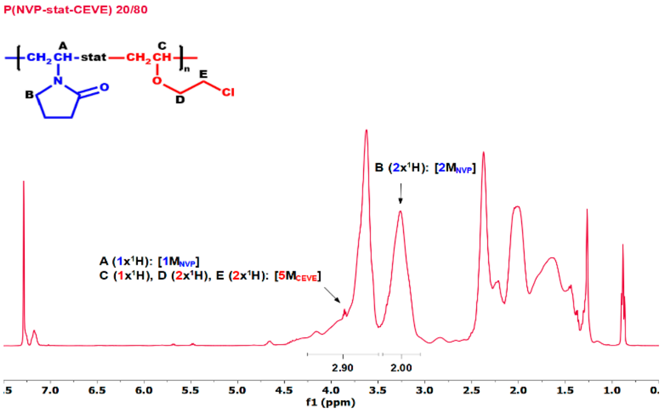

(e)

Figure S2. Representative ^{1}H-NMR spectra in $CDCl_3$ of the P(NVP-stat-CEVE) statistical copolymers: **(a)** 80/20; **(b)** 60/40; **(c)** 50/50; **(d)** 40/60; and **(e)** 20/80. The copolymer actual composition was calculated from the portions of characteristic signals assigned to the NVP and CEVE monomer units, utilizing portions of protons **A** and **B** for **NVP**, and portions of protons **C**, **D**, and **E** for **CEVE**, as shown in the spectra.

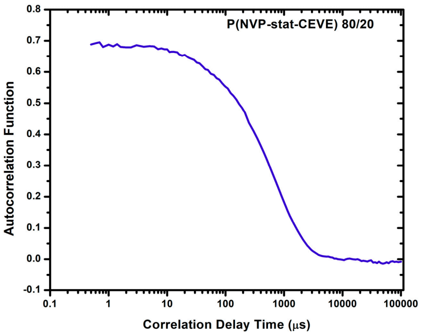

(**a**)

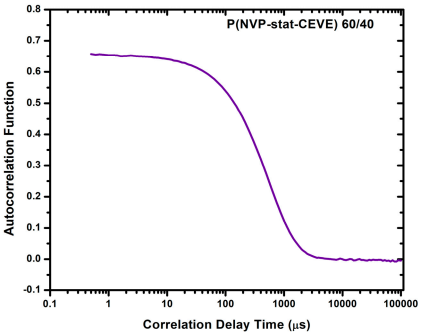

(**b**)

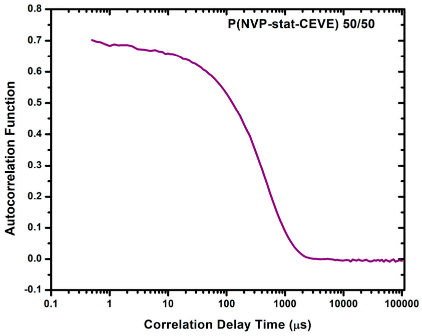

(**c**)

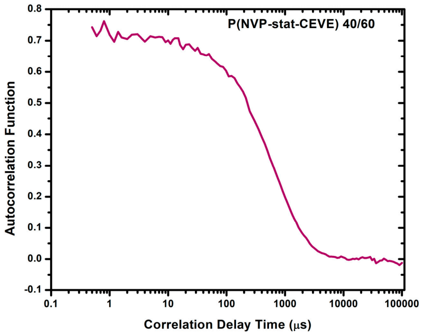

(**d**)

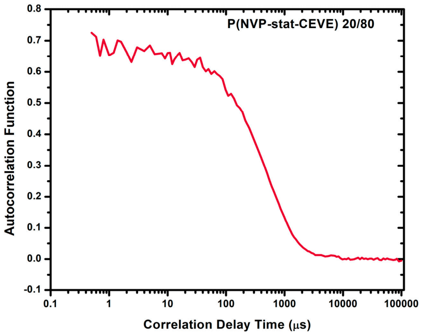

(e)

Figure S3. Intensity time autocorrelation functions derived from Dynamic Light Scattering (DLS) measurements in aqueous media for the P(NVP-stat-CEVE) statistical copolymers: **(a)** 80/20 C = 0.462 mg/mL; **(b)** 60/40 at C = 0.324 mg/mL; **(c)** 50/50 at C = 0.492 mg/mL; **(d)** 40/60 at C = 0.204 mg/mL; and **(e)** 20/80 at C = 0.226 mg/mL.

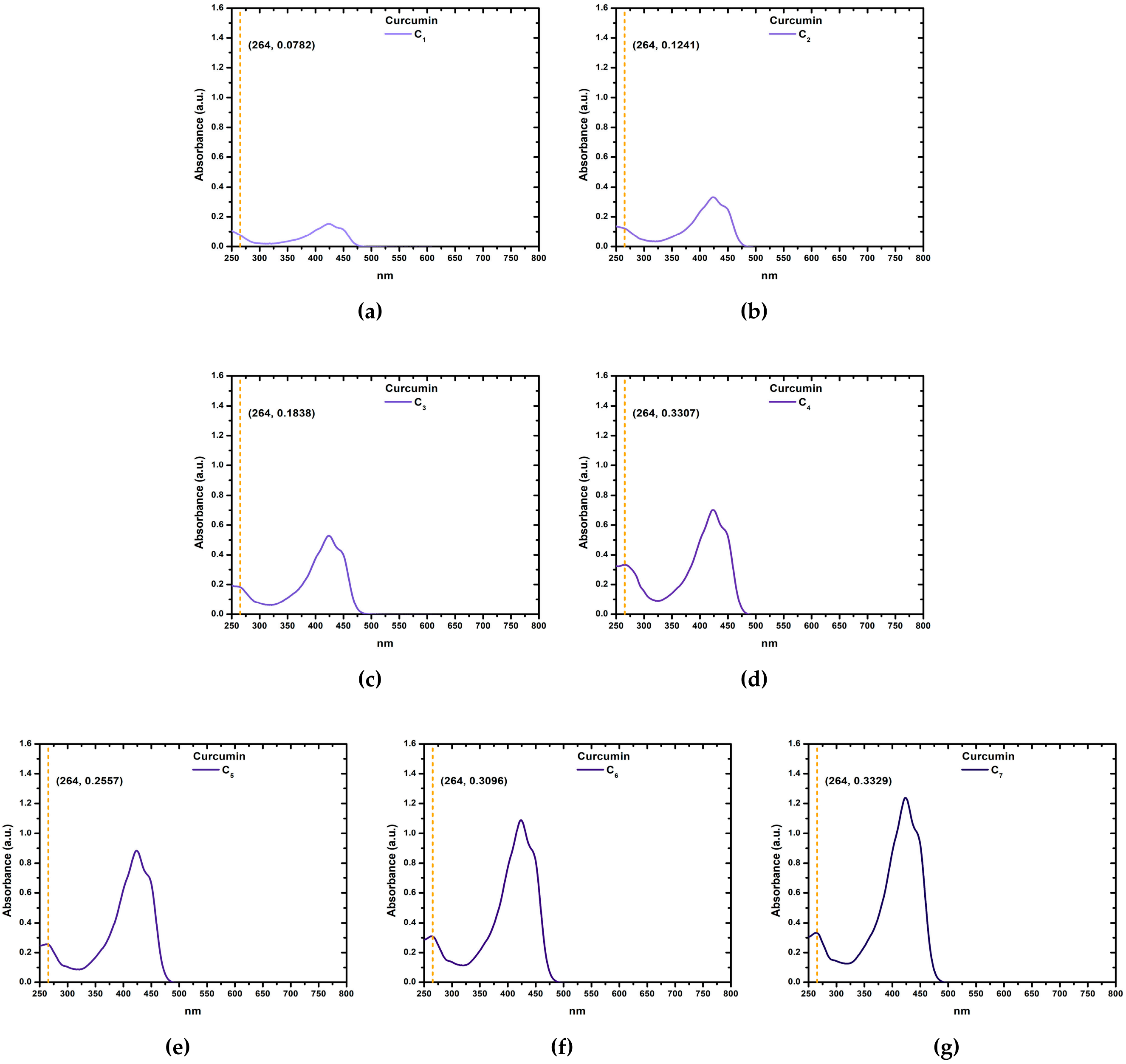

Figure S4. Individual UV-Vis absorption spectra of curcumin in chloroform ($CHCl_3$) corresponding to the seven prepared concentration levels used for the calibration process: **(a)** C_1 = 0.52 μg/mL; **(b)** C_2 = 1.40 μg/mL; **(c)** C_3 = 2.36 μg/mL; **(d)** C_4 = 3.28 μg/mL; **(e)** C_5 = 4.23 μg/mL; **(f)** C_6 = 5.25 μg/mL; and **(g)** C_7 = 6.10 μg/mL. The dashed vertical line denotes the theoretical absorption maximum of the diketo tautomer at 264 nm.

Table S1. Concentration and corresponding absorbance values of curcumin in chloroform at λ_{max} = 264 nm, utilized for the construction of the calibration curve.

**Sample**	**C_1** *	**C_2**	**C_3**	**C_4** *	**C_5**	**C_6**	**C_7**
Concentration (µg/mL)	0.52	1.40	2.36	3.28	4.23	5.25	6.10
Absorbance (a.u.)	0.0482	0.1241	0.1838	0.3307	0.2557	0.3096	0.3329

* Samples C_1 and C_4 (highlighted in red) were excluded from the linear regression analysis. Sample C_1 was rejected because it failed to form a distinct, normal peak; the recorded absorbance value fell on the spectral slope, rendering it an invalid data point. Sample C_4 was discarded due to an evident experimental error, as it exhibited an anomalously higher absorbance than the subsequent samples (C_5 and C_6) despite having a lower concentration.

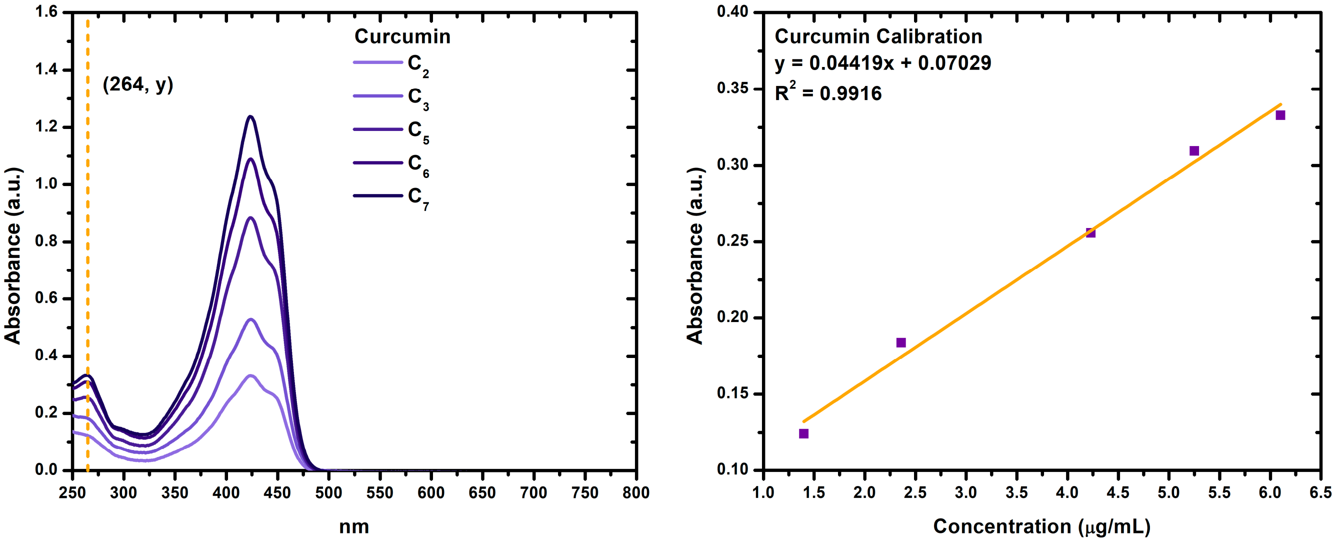

(**a**) (**b**)

Figure S5. Construction of the curcumin calibration curve in chloroform: (a) Overlay of the UV-Vis absorption spectra for the five valid concentration levels (C_2, C_3, C_5, C_6, and C_7); (b) The resulting linear calibration curve plotting absorbance at 264 nm versus curcumin concentration, exhibiting a high correlation coefficient (R^2 = 0.9916).

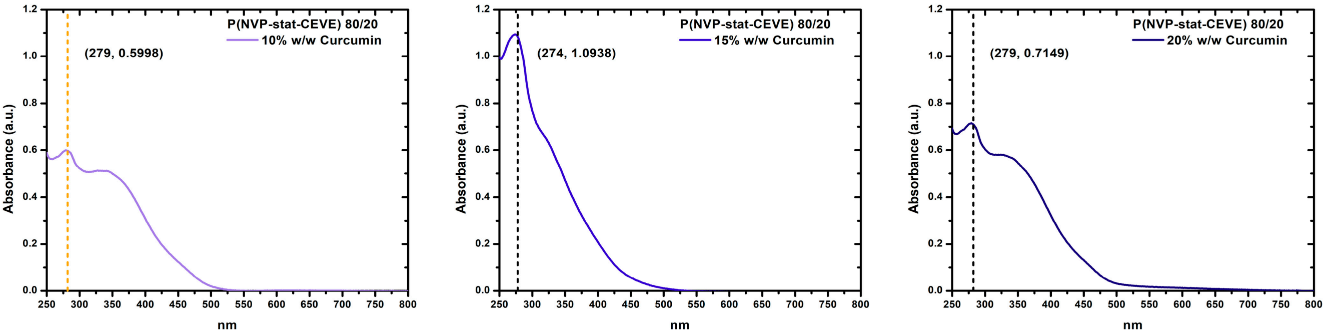

(a) **(b)** **(c)**

Figure S6. Isolated UV-Vis absorption spectra of the encapsulated curcumin within the aqueous micellar solutions of the P(NVP-stat-CEVE) 80/20 sample at three initial drug-to-polymer feed ratios: **(a)** 10% w/w; **(b)** 15% w/w; and **(c)** 20% w/w. The dashed vertical line denotes the characteristic absorption maximum of the encapsulated diketo tautomer.

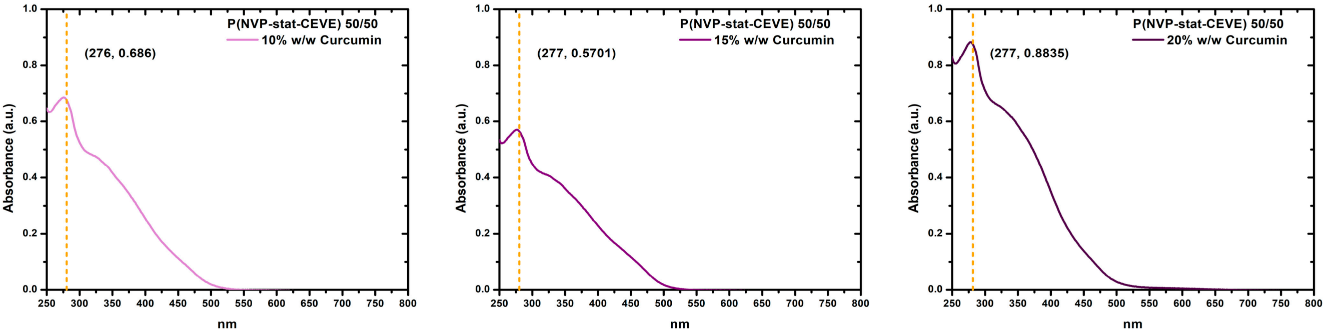

(a) **(b)** **(b)**

Figure S7. Isolated UV-Vis absorption spectra of the encapsulated curcumin within the aqueous micellar solutions of the P(NVP-stat-CEVE) 50/50 sample at three initial drug-to-polymer feed ratios: **(a)** 10% w/w; **(b)** 15% w/w; and **(c)** 20% w/w. The dashed vertical line denotes the characteristic absorption maximum of the encapsulated diketo tautomer.

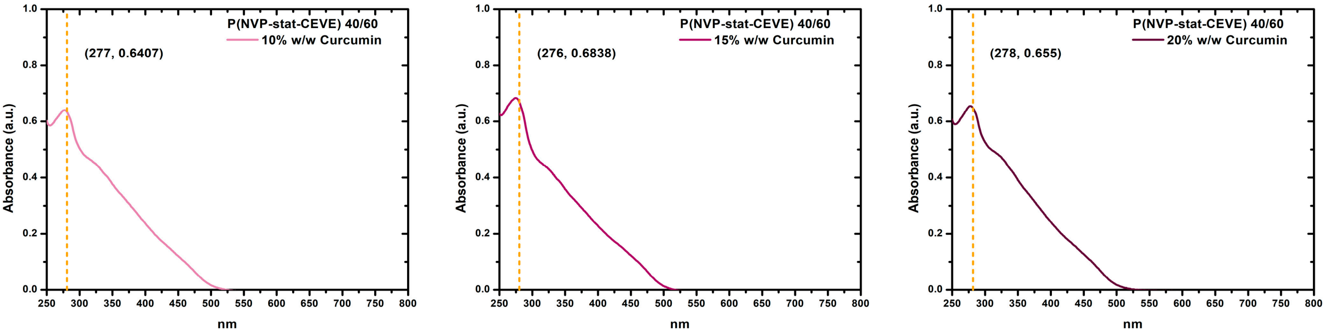

(a) **(b)** **(c)**

Figure S8. Isolated UV-Vis absorption spectra of the encapsulated curcumin within the aqueous micellar solutions of the P(NVP-stat-CEVE) 40/60 sample at three initial drug-to-polymer feed ratios: **(a)** 10% w/w; **(b)** 15% w/w; and **(c)** 20% w/w. The dashed vertical line denotes the characteristic absorption maximum of the encapsulated diketo tautomer.

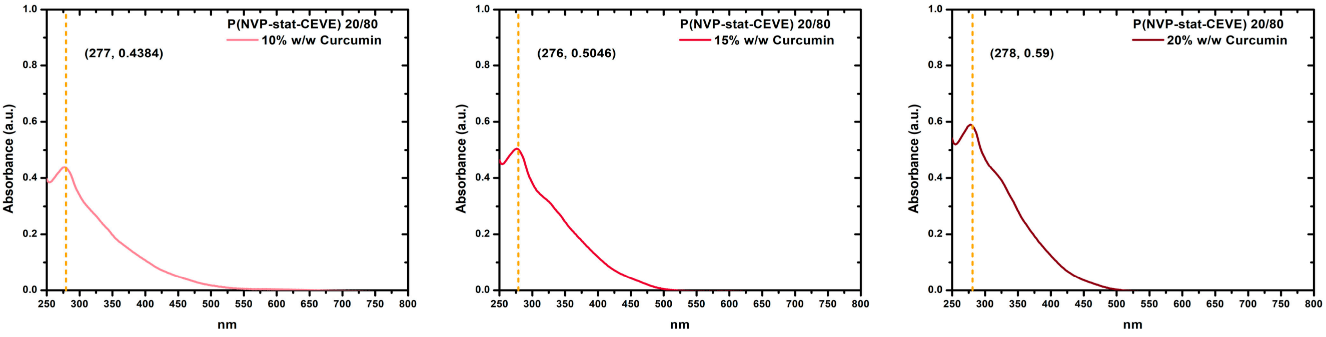

(a) **(b)** **(c)**

Figure S9. Isolated UV-Vis absorption spectra of the encapsulated curcumin within the aqueous micellar solutions of the P(NVP-stat-CEVE) 20/80 sample at three initial drug-to-polymer feed ratios: **(a)** 10% w/w; **(b)** 15% w/w; and **(c)** 20% w/w. The dashed vertical line denotes the characteristic absorption maximum of the encapsulated diketo tautomer.
